# Dentistry responding to domestic violence and abuse: a dental, practice-based intervention and a feasibility study for a cluster randomised trial

**DOI:** 10.1038/s41415-022-5271-x

**Published:** 2022-12-09

**Authors:** Paul Coulthard, Gene Feder, Maggie A. Evans, Medina Johnson, Tanya Walsh, Peter G. Robinson, Christopher J. Armitage, Estela Barbosa, Martin Tickle, Omolade Femi-Ajao

**Affiliations:** 4141534059001 Queen Mary University of London, London, E1 2AD, UK; 4141534059002 University of Bristol, Bristol, BS8 2PS, UK; 4141534059003 Chief Executive, IRISi, BS8 2PS, UK; 4141534059004 University of Manchester, Manchester, M13 9WL, UK; 4141534059005 University of Bristol Dental Hospital, Bristol, BS1 2LY, UK; 4141534059006 Manchester University NHS Foundation Trust, National Institute for Health and Care Research Greater Manchester Patient Safety Translational Research Centre, University of Manchester, M13 9WL, UK; 4141534059007 University College London, London, EC1V 0HB, UK

## Abstract

**Objectives ** Assess the feasibility of using the Identification and Referral to Improve Safety (IRIS) intervention ina general dental practice setting and evaluating it using a cluster randomised trial design. IRIS is currently used in general medical practices to aid recognition and support referral into specialist support of adults presenting with injuries and other presenting factors that might have resulted from domestic violence and abuse. Also, to explore the feasibility of a cluster randomised trial design to evaluate the adapted IRIS.

**Design**Feasibility study for a cluster randomised trial of a practice-based intervention.

**Setting ** Greater Manchester general dental practices.

**Results ** It was feasible to adapt the IRIS intervention used in general medical practices to general dental practices in terms of training the clinical team and establishing a direct referral pathway to a designated advocate educator. General dental practices were keen to adopt the intervention, discuss with patients when presented with the opportunity and utilise the referral pathway. However, we could not use practice IT software prompts and data collection as for general practitioners because there is no unified dental IT system and because coding in dentistry for diagnoses, procedures and outcomes is not developed in the UK.

**Conclusion ** While it was feasible to adapt elements of the IRIS intervention to general dental practice and there was general acceptability, we did not have enough empirical data to plan a definitive cluster randomised trial design to evaluate the IRIS-dentistry intervention within general dental practices.

## Key points

- Describes the process of developing an intervention used in general medical practices for the identification and referral of patients experiencing domestic violence and abuse to advocacy services for use in general dental practices.
- An adapted intervention was generally acceptable to general dental practice staff and resulted in a high level of engagement.
- There are no unified IT systems in general dental practices in the UK as there are in general medical practices and so it was not possible to provide prompts to the staff or collect data digitally for analysis. The collection of sufficient data for the planning of a large definitive study was not possible.

## Introduction

Domestic violence and abuse (DVA) takes place between individuals in intimate relationships and between adult family members and may manifest as physical violence, sexual abuse, financial abuse, psychological abuse and/or controlling behaviour.^1^ It is a violation of human rights with short- and long-term consequences for physical and mental health. The COVID-19 pandemic has highlighted DVA and the increased risk brought about by household isolation.^2^

When DVA involves physical assault, the face is a very common target, with studies suggesting that between 65-95% of assaults involve trauma to the face, mouth and teeth.^3^^,^^4^^,^^5^^,^^6^ Consequently, the dentist, dental care professional, oral surgeon and the oral and maxillofacial surgeon all have a critical role to play in identifying DVA.^7^^,^^8^

There are approximately 15.4 million incidents of DVA annually in Britain.^1^ Aside from the physical and psychological harm, the total annual monetary cost of DVA in England for the government, the individual (human and emotional suffering), the NHS, employers and the criminal justice system is around £23 billion. An estimated 2.0 million UK adults aged 16-59 years experienced domestic abuse in the year ending March 2018, equating to a prevalence rate of approximately 6 in 100 adults. Women were around twice as likely to experience domestic abuse as men (7.9% compared to 4.2%).^9^

Policy frameworks and National Institute for Health and Care Excellence quality standards around DVA often refer to 'all healthcare professionals' being involved in identifying, supporting and referring to specialist advocacy services.^10^ However, dentistry is not often explicitly mentioned and implementation in dental services has been low. There is very limited specific dentistry DVA training and referral pathways.

A lack of training, the presence of a patient's partner or children, concern about offending patients, funding restrictions, limited use of IT and dentists' own embarrassment about raising the topic of abuse are important barriers to identifying, referring and supporting victims of DVA within dental services.^11^ There may also be misconceptions and lack of awareness about the role of dentists and dental care professionals in supporting patients experiencing DVA.^12^ Furthermore, there is a dearth of research on the effectiveness of domestic violence interventions within dentistry.^13^

An evidence-based programme of practice training and a referral pathway to DVA advocacy has been developed with general medical practices (GMPs) and is commissioned in over 40 areas nationally.^14^^,^^15^^,^^16^ The Identification and Referral to Improve Safety (IRIS)care pathway has been widely used within GMPs to identify and support patients experiencing DVA and has been shown to improve identification and referral of victims and survivors to appropriate specialist support agencies.^16^^,^^17^^,^^18^^,^^19^ It has also been shown to be cost-effective.^20^ However, this care pathway has never been used within general dental practices (GDPs). IRIS cannot be directly translated to GDPs due to some significant differences between the dental and medical practice settings. In this paper, we report the feasibility of using a similar practice-based intervention (IRIS) for use to support GDPs.

The IRIS intervention comprises:

- Practice-based DVA training sessions for clinical and administrative staff
- Installation of software to prompt enquiring and recording of DVA information
- A referral pathway to a named DVA advocate, who also delivers training and supports victims to contact appropriate DVA services.

Across England, 28.9 million patients are seen by an NHS dentist, costing about £4 billion annually.^21^ A high proportion of NHS practices are 'mixed', with some care provided independently of the NHS payment system and there are also dental practices that are entirely private. Some of these patients have been reported to present with injuries resulting from domestic violence within general dental practices.^16^ Work of Coulthard and Warburton (2007) on the role of the dental team in responding to DVA highlighted the unique characteristics of the dental practice in offering victims the space and privacy to disclose abuse without interference from the perpetrator.^22^ Dental personnel are in a unique position to recognise, document and refer such victims and survivors of domestic violence for appropriate assistance.

We wanted to determine whether an IRIS-type care pathway in dentistry was feasible. The research had two aims:

- Assess the feasibility of adapting the IRIS intervention to aid recognition and support the referral of adults presenting in a primary care dental setting with injuries that might have resulted from DVA
- Explore the feasibility of a cluster randomised trial design to evaluate the adapted IRIS intervention in dental practices.

The objectives relating to aim one were:

- Adapt a GMP DVA training and support intervention for the GDP context
- Assess the level of engagement of practice-based staff with regards to training and support
- Examine the acceptability of IRIS intervention within primary care dental practices with a nested qualitative study.

The objectives relating to aim two were:

- Measure recruitment rates and examine reasons for attrition/non-participation
- Establish the suitability of the primary and secondary outcome measures and test the feasibility of data collection for a trial in primary care dental practices. Primary outcome = number of referrals to DVA advocate for nine months after training of intervention practices. Secondary outcome = DVA disclosure rate for nine months after training of intervention practices
- Document the types of dental injuries resulting from DVA
- Articulate health economics costs that can be measured directly and ensure robust collection of these costs to inform the cost-effectiveness analysis in a definitive trial
- Estimate sample size for an adequately powered definitive trial.

## Methods

### Design and sampling strategy

This cluster randomised feasibility study selected GDPs as the unit of randomisation, identified through the Greater Manchester Dental Professional Network. These practices reflected a range of geographic sites/socioeconomic indicators and a mix of NHS and private practice. The study took place over 24 months, with an additional three-month non-cost extension.

The intervention involved implementing the different components of the IRIS care pathway by training staff in GDPs and providing a link to a designated advocate educator from the collaborating agency, Manchester Women's Aid's IRIS team.

### Development phase

We worked with a range of stakeholder's and a patient and public involvement (PPI) group, including victims and survivors of DVA, Manchester Women's Aid, Trafford Domestic Abuse Service and Mankind, as well as dentists, dental care professionals and practice administrative staff over six months. This development group of ten individuals adapted the training delivered during the feasibility study. This group size provided reasonable representation of the stakeholders. The research team prepared draft intervention models and the group met over three workshops to finalise the IRIS-dentistry intervention. Using the Behaviour Change Wheel,^23^ the methodology adopted for the development phase corresponded with the updated Medical Research Council framework for complex interventions.

**Fig. 1:**
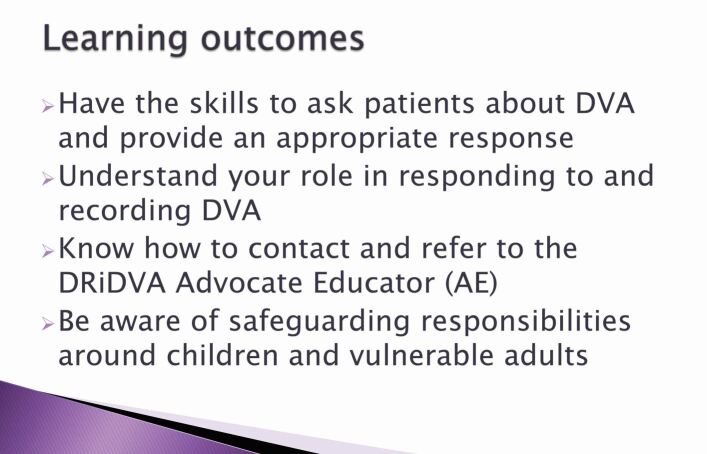
Example slide from the training presentation

**Fig. 2:**
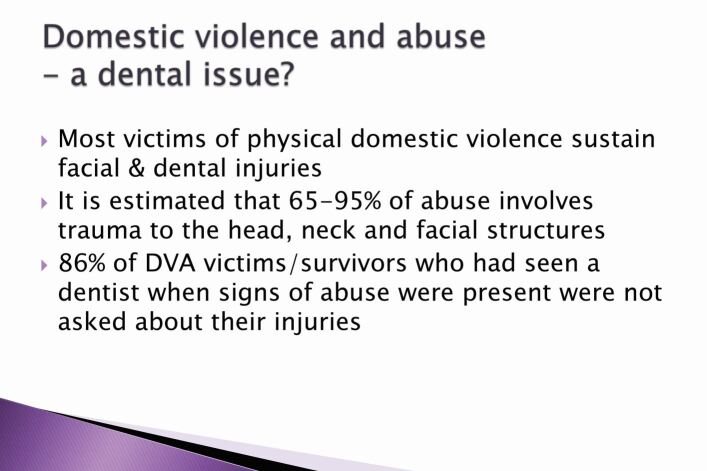
Example slide from the training presentation

Example slides from the training presentation are shown in Figures 1 and 2.

### Recruitment, training, data collection and follow-up

We recruited six GDPs between June and July 2018, based on evidence from Eldridge *et al*.,^24^ against using information from feasibility studies alone to estimate sample sizes for full cluster randomised controlled trials.

We aimed to train all practice staff (dentists, dental nurses, practice manager, hygienist, safeguarding lead) in the intervention practices, taking into consideration peer influence and group reinforcement.

Three practices were randomly allocated to the intervention arm and three to the usual practice (no intervention) arm, using block randomisation. Practice principals were provided with a participation information sheet and consent was obtained. The practice-level training was delivered in the GDP by the advocate educator from Manchester Women's Aid's IRIS team, together with a dentist; the study principal investigator (PC). This dual approach is a key component of the IRIS intervention. Clinical training session one was a two-hour training session delivered over two one-hour sessions, and clinical training session two was one-hour training delivered six weeks after the second hour clinical session one training. The sessions were arranged at lunchtimes and lunch was provided.

Over the course of the study period, additional training was delivered to reinforce the intervention and enhance staff capacity. In addition, during the non-cost extension period (September to October 2019), additional training was delivered to newly employed staff in the intervention practices. The comparator, usual practice (no intervention) GDPs were asked to follow their usual practice for safeguarding high-risk and vulnerable adult patients.

We aimed to collect the following data: date disclosure of DVA; age; sex; type of injury resulting from DVA; and whether a referral was made. All six practices were given anonymised paper-copy forms to capture data. These forms were necessary as there were no unified IT systems within the practices to facilitate electronic data capture. The data forms were then collected weekly from the manager at each practice by the project coordinator (OF-A).

### Nested qualitative study

During follow-up, qualitative interviews explored the adherence to and acceptability of the IRIS intervention and study design. Individual interviews were conducted with dentists from the three intervention practices that attended the clinical session one and clinical session two training. Three focus group discussions also took place with dental care professionals and reception and practice management staff at the intervention practices.

### Intervention development for dental practice context

The IRIS intervention for general medical practices consists of:

- Practice-based DVA training sessions for clinical and administrative staff
- Installation of software to prompt enquiring and recording of DVA information
- A referral pathway to a named DVA advocate, who also delivers training and supports victims to contact appropriate DVA services.

The training package for GDP was developed by the PPI group, advocate educators, dentists, dental care professionals (hygienists, therapists and nurses), receptionists and practice managers over three stakeholder workshops. The standard IRIS PowerPoint presentation was modified to include 'domestic violence and abuse - a dental issue?', with a section providing facts and figures and illustrated clinical cases of dental injury. A video was produced in the dental setting illustrating 'how to' and 'how not to' facilitate disclosure of DVA and how to respond and manage a referral. Stakeholder workshops also discussed the relevant differences of GDPs to GMPs.

The modified IRIS training was delivered by the advocate educator and a dentist (PC) in September and November 2018. Clinical session one was a two-hour training session delivered over two visits, and clinical session two was a one-hour training session delivered six weeks after the second hour of the clinical session one delivery.

### Willingness of general dental practices to be randomised

All the six dental practices approached provided written informed consent to be randomised to the study to intervention or usual practice (no intervention).

### Feasibility of manually collecting identification referral data from dental records via dental team members

Different systems were being used to record patient consultation in the three intervention practices. Two used paper notes, with the electronic IT software used for other functions, such as patient bookings. Weekly visits were made to each intervention practice to collect the anonymised paper-copy data study collection forms.

### Acceptability of the modified IRIS intervention

Focus groups and individual qualitative interviews were conducted after the completion of all clinical session two training. The selection criteria for study participants was informed by whether the dentists had made a referral, their years of experience (younger dentist/older dentist) and whether they worked full-time or part-time, with the intention of having a diverse group. All study participants in the qualitative interviews had attended at least two of the three hours of training. Focused group discussion sessions were held with dental nurses and reception staff across the three dental practices in the intervention arm.

### Feasibility of a definitive cluster randomised trial design

We looked at the feasibility of undertaking a fully powered cluster randomised trial to evaluate the adapted IRIS in GDPs. We planned to satisfy the following objectives:

- Measure recruitment rates and examine reasons for attrition/non-participation
- Establish the suitability of the primary and second outcome measures and test the feasibility of data collection for a trial in primary care dental practices
- Document the types of dental injuries observed as resulting from DVA
- Articulate health economics costs that can be measured directly and ensure robust collection of these costs to inform the cost-effectiveness analysis in a definitive trial
- Estimate sample size for an adequately powered definitive trial.

## Results relating to feasibility of using an adapted iris intervention in dental pracitices

### Intervention development for GDPs

We approached six GDPs and all consented to recruitment for this feasibility study, of which three participated in the modified IRIS intervention for GDPs.

The staff training is delivered by an advocate educator and general practitioner duo in the IRIS model and we replicated this with an advocate-educator and dentist duo. We were not, however, able install software in the electronic medical system of the three dental practices.

One practice used Software of Excellence (SOE), another Bridge-IT and the other, R4 Software. SOE permits free-text search. The other types of software used do not permit free-text search. The practice using R4 noted that it was not possible to enter information about DVA and that concerns would be discussed with the practice manager who was the safeguarding lead for the practice.

### Level of engagement of practice staff

In total, 16 dentists and 23 dental care professionals were trained across three dental practices. This was all of the members of the dental teams at these practices. The modified IRIS training was delivered by the advocate educator and dentist (PC) as planned, in September and November 2018. Clinical session one was a two-hour training session delivered over two visits, and clinical session two, was a one-hour training session delivered six weeks after the second hour of clinical session one delivery.

Not all staff attended all training sessions or all of each one. Some staff (three dentists) attended only the first hour of clinical session one and not the second part, and did not attend clinical session two. Similarly, some staff (four dentists and seven dental care professionals) attended the second part of clinical session one but not the first part, and did not attend clinical session two. Only nine dentists and 16 dental nurses attended the full clinical session training. Two reception staff and one practice manager also attended the three hours of training. Additional one-hour training was delivered in September 2019 for newly employed staff in the three intervention practices. In total, seven dentists and nine dental nurses received the one-hour training in September 2019.

### Focus groups and individual qualitative interviews

In total, there were three focus groups and nine individual interviews. Underlying themes were identified from five selected transcripts by two study investigators (MAE, OF-A) independently and these were used for the analysis (Table 1.) Example, paraphrased reflections are reported in Box 1.

**Table 1:** Qualitative interviews identified underlying themes

Theme	Outcome
Clinician underlying anxiety	Training reduced clinician anxiety
Patients' experience	Clinicians are able to understand
Timing of training sessions	Important to schedule at a time of best convenience for staff
Behaviour change	Training led to an increased awareness of DVA, increased knowledge of DVA and positive attitude
Tipping point for patient disclosure of DVA	Clinicians' understanding increased
Training sessions time	Requested reduced time and/or combine two sessions into a single session
Training reinforcement	Request for regular updates
DVA awareness and knowledge	Clinicians' increased awareness and knowledge
Safe environment	Clinicians' recognised utility of dental surgery for disclosure
Named advocate educator	Not necessarily important

Stakeholder workshops also discussed the relevant differences of GDPs to GMPs. These were: no unified IT system in dental practices; payment systems' time for training constraints; patients are customers and are a mix of NHS and private; corporate practices; more staff in attendance with patients, private space?; building trust and confidence so that disclosure can be made; and some preference for no waiting area posters about DVA.

Box 1Example paraphrased comments from qualitative interviews

1. Dentist had a conversation with a patient as part of a clinical enquiry about their dental trauma. The patient disclosed it was from domestic abuse but she was already in touch with social services, as her kids had been removed. No further action with respect to DVA was undertaken by the dentist
2. Dentists had a conversation about the dental trauma a female patient presented with. The clinician perceived that the explanation the patient gave was inconsistent with the injuries and asked the patient whether the injury was due to DVA. The patient disclosed that she was experiencing DVA. Referral was made to the named advocate educator for the study
3. A dental nurse shared a previous experience of having a patient disclose they were experiencing domestic abuse. The dental nurse recorded this information and passed it on to the advocate educator
4. Two full-time dentists had conversations with patients about domestic abuse. The conversations were informed by the facial injuries the patients had sustained. Both patients said their injuries were not as a result of domestic abuse
5. A patient attended with a black eye and had attended a few weeks earlier with bruising to the cheek and said she had fallen. The reception team who had received the training booked the patient in and then informed the dentist of their DVA concerns. The dentist then had a conversation with the patient who denied DVA but accepted the phone contact for the advocate educator when offered

## Results relating to feasibility of a definitive cluster randomised trial

### Willingness of GDPs to be randomised

All six dental practices provided written informed consent to be randomised to the study to intervention or usual practice (no intervention). There were no issues with the randomisation. All six practices were offered a small research expense fee. There was limited contract with the three 'usual practice' sites; however, all declined accepting this fee. The principal of one of these practices commented that he had never come across patients with DVA experience in his dental practice, that is, no suspicious or unexplained dental injury and no disclosures.

### DVA identification and referral data capture

Between September 2018 and October 2019, clinical staff across the three dental practices had domestic violence-focused enquiries (conversations) with 11 adult patients who had presented with injuries that were suspected to have been as a result of domestic abuse. Ten patients were women and one was a man. Of the eleven patients, four disclosed historical cases of domestic abuse. Two patients were referred to the named advocate educator. Although some of the identified patients could not be directed or supported as part of this study, all patients were given the contact details of the DVA advocate educator.

In addition, practice managers from the intervention practices reported that patients regularly took the contact details of the advocate educator from tear-off strips on study posters that had been placed in the practice patient toilets.

### Feasibility of a definitive cluster randomised trial design

We looked at the feasibility of undertaking a fully powered cluster randomised trial to evaluate the adapted IRIS in GDPs. We were unable to satisfy our objectives, as we did not collect sufficient quantitative data. However, findings from our qualitative study and the narrative provided by the advocate educator suggests that the research has started the process that may, potentially, change current practice and service provision within dentistry for adult patients with lived experience of domestic abuse.

We were unable to calculate health economics costs to inform the cost-effectiveness analysis in a definitive trial, as we did not collect sufficient empirical data. We did, however, estimate excess treatment costs involved in identification and referral of an individual patient to constitute 30-minute patient contact instead of a ten-minute check-up, that is, 20 minutes of additional time, equivalent to two lost units of dental activity: 2x (£28 + on-costs) = 2 x £33 = £66 per patient. Applying this to three study intervention practices over the course of 12 months with one patient identified per month would equate to a cost of £2,376.00. Training time would also need to be considered.

## Discussion

### Intervention

The staff training was delivered by an advocate educator and general practitioner duo in the IRIS model and we replicated this with an advocate educator and dentist duo. This is seen to be an important feature of the intervention, likely because the doctor or dentist is persuaded that the DVA role is an important aspect of their professional responsibility. We also established a referral pathway to a named advocate educator from the collaborating agency (Manchester Women's Aid).

We were not able install software in the electronic medical system of the three dental practices. Prompts for the dental team members could not be used, as IT systems in GDPs are not unified. Similarly, software used by dental practices for record keeping couldn't be used for study data collection as these are not unified and also because coding is not developed. Coding for dental procedures, disease or condition (for example, trauma) and clinical patient recorded outcome measures is embryonic in the UK. This severely limited our ability to collect meaningful data effectively. One practice used SOE, another Bridge-IT, and the other, R4 Software. The lack of unified systems for recording DVA to allow data collection is a significant problem for dentistry. We understand that NHS Digital is committed to introducing a novel and consistent coding system across primary healthcare, including dental practice, in both the NHS and private GDPs.

### Staff engagement

Not all staff attended all training sessions or all of each one. The practices were members of a research network and had agreed to take part and were being remunerated by National Institute for Health and Care Research (NIHR) to do so. It may be that staff did not value the intervention sufficiently, were not paid enough, or perhaps the sessions were not scheduled at convenient times. Clinical dentistry cannot be expected to run perfectly to time and inevitably there is unpredictability that impacts on training sessions booked over lunchtime. There may be holiday and sickness that also prevents attendance. Alternatively, it may have reflected the absence of continuing professional development reward, or another reason. In general, we had good engagement.

### Focus groups and individual qualitative interviews

The qualitative data showed that study participants found the modified IRIS intervention very acceptable and that attending the training raised their awareness of domestic abuse among the general population and among their adult patient population. Participants volunteered that the training empowered them to be able to have conversations with patients about DVA that they previously would not have had. They were now aware of DVA signs to look out for and had an improved level of confidence, such that they could speak in a reassuring manner with the patient about DVA. They reported that they understood and were confident of the referral pathway to the DVA advocate.

There were mixed views about the pertinent role and importance of having a named DVA advocator, who was the same individual who provided the training, someone known to the dental practice, as a key indicator in making a referral and the subsequent uptake of a referral. Some study participants suggested that having a named advocate educator known to the dental practice may not necessarily influence them to make a referral to the collaborating agency, as what was more important to them was knowing what to do when a patient discloses domestic abuse during a dental appointment consultation.

Not all study participants had made a referral to the advocate educator during the follow-up period. Staff members who had used the referral pathway reported that being trained and having met the named advocate educator were crucial factors in their response. For study participants who were yet to have a conversation with patients or use the referral pathway, they reported concerns, such as the need for more professional experience and the amount of paperwork that could potentially be involved in making a referral. All study participants identified the need for reinforcement to enable them continue to engage with the subject area. The suggested duration of interval between reinforcement ranged from often to once in six months.

### Willingness of GDPs to be randomised

All six dental practices provided written informed consent to be randomised to the study to intervention or usual practice (no intervention). It is of note that the principal of one of the practices randomised to 'usual practice' denied the likelihood of any patient attending with injury caused by DVA.

### Planning of definitive study

We looked at the feasibility of undertaking a fully powered cluster randomised trial to evaluate the adapted IRIS in GDPs. While we recruited six GDPs for this feasibility study, the parameters used in deciding the sample size of six GDPs for the definitive trial would include: information obtained from our research; taking into consideration the variation of settings (for example, practice size, location, etc); and comparable evidence from cluster randomised trials in primary medical care, to aid in estimating the intracluster correlation coefficient.

The size of the cluster would vary, depending on the number of staff in the practices. However, we envisaged that there would be a minimum of six participants (two dentists, one practice manager, one reception staff, one dental nurse/hygienist and one practice safeguarding lead) per cluster.

Our objectives were those of 'objectives two' above, that include measurement of recruitment rates, testing the feasibility of data collection for a trial in primary care dental practices, articulating the health economics costs that can be measured directly and estimating the sample size for an adequately powered definitive trial. We were unable to satisfy these objectives, as we did not collect sufficient quantitative data. Since the overarching aim of this research was to explore the feasibility of developing a training and support practice-level intervention in improving the health and social care outcomes of adult patients with lived experience of DVA utilising primary care dental practices, it was not our intention to measure or demonstrate impact. However, findings from our qualitative study and the narrative provided by the advocate educator suggests that the research has started the process, which may potentially change current practice and service provision within dentistry for adult patients with lived experience of domestic abuse.

## Conclusion

We found that it was feasible to adapt elements of the IRIS intervention used in GMPs for a GDP setting, in terms of the training presentation and establishing a referral pathway to a designated advocate educator. We were not, however, able to use dental practice IT software prompts and data collection as for general practitioners because there is no unified IT system and also because coding in dentistry is not developed.

We also have evidence from the feasibility study to show that GDPs were keen to adopt the intervention, staff felt empowered to have conversations with patients when presented with the opportunity that they previously would not have had, and were confident and able to utilise a referral pathway to a DVA advocate. Practice managers from the intervention practices reported that patients regularly took the contact details of the advocate educator from tear-off strips on study posters that had been placed in the practice patient toilets.

However, we were unable to resolve all the existing uncertainties documented in the objectives of the feasibility studies to plan a definitive large cluster randomised trial design to evaluate the IRIS-dentistry intervention within the primary care dental setting.
